# The effect of different parenting styles on the child behavior during the dental visit: observational longitudinal study

**DOI:** 10.1186/s12903-025-05659-2

**Published:** 2025-03-05

**Authors:** Sara Saiid Henedy, Amany M. Khalil, Sawsan Hafez Mahmoud, Laila Moustafa El-Habashy

**Affiliations:** 1 Pediatric Dentistry and Dental Public Health Department, Faculty of Dentistry, Alexandria University, Alexandria, Egypt; 2 Pediatric Dentistry and Dental Public Health Department, Faculty of Dentistry, Pharos University, Alexandria, Egypt

**Keywords:** Child Dental Behavior, Parenting style

## Abstract

**Background:**

It is widely recognized that parental influence significantly shapes children’s emotional health and behavioral patterns. The current study aims to evaluate the effect of different parenting styles on the child’s behavior during the dental visit.

**Methods:**

Sixty healthy children age ranging from 4 to 6 years old who needed class I restoration in one of their primary molars were selected from the outpatient clinic of the Pediatric Dentistry and Dental Public Health Department, Faculty of Dentistry, Alexandria University, Egypt. Both parents signed a consent form to participate in the research and filled out the study questionnaires. Two questionnaires were used in this study: the Parenting Styles and Dimensions Questionnaire (**PSDQ**) and the demographic questionnaire to determine the parents’ socioeconomic status. The Frankl rating scale was used to evaluate the children’s behavior during the initial examination visit and the subsequent restorative visit. The procedure time was assessed from the videotaped restorative visit.

**Results:**

The overall child behavior during the examination visit and the second restorative visit showed that children of authoritative parents demonstrated significantly positive behavior (Frankl rating 3) compared to the negative behavior in children of both authoritarian and permissive parents (Frankl rating 2) (*P* = 0.002, *P* = 0.004). The authoritative parenting style showed significantly less operative time than the authoritarian and permissive groups (*P* = 0.001, 0.008, respectively).

**Conclusion:**

Children of authoritative parenting style showed significantly positive child behavior on the dental chair than those of permissive and authoritarian parenting styles.

**Supplementary Information:**

The online version contains supplementary material available at 10.1186/s12903-025-05659-2.

## Background

One of the most challenging circumstances for pediatric dentists to provide efficient dental treatment is children’s uncooperative behavior; it can result in difficulty performing the dental treatment and unsatisfactory outcomes. Additionally, bad behavior might cause a delay in receiving critical dental care, allowing the condition to progress and necessitating the use of more advanced techniques like sedation and general anesthesia [1].

Recently, researchers have studied parenting styles with great interest. A parenting style is similar to the emotional environment that arises from interactions between parents and children; this environment can be deduced from the way that parents engage with support, monitor, and react to their kids [2]. The family environment significantly influences the daily patterns of behavior in children. According to Darling et al., parental styles have a direct impact on children’s behavior, which in turn modifies the link between parental expectations and child outcomes, leading to an indirect impact on the child’s progress and development [3].

Baumrind asserts that parenting practices affect a child’s general social development, competence, accomplishment, and cognitive growth, which is a major factor that can influence how a child behaves in the field of dentistry [4]. Additionally, the manner in which a child manages tension and stimuli, such as those encountered in a dental environment, is also influenced by their parenting approach. Numerous studies have demonstrated a direct correlation between dental stress tolerance in children and coping skills [5–7].

Baumrind has identified three parenting approaches frequently referenced in literature: authoritative, authoritarian, and permissive [8]. The authoritative parenting style (high warmth, high control) is defined by nurturing warmth and emotional support while continuing to enforce strict control on the child’s behavior. The authoritative parenting style is logical and always supports and encourages the child continuously; it relies less on giving orders and more on reasoning and explanations. These parents typically communicate with their children in both directions, which develops and preserves the parent-child relationship [9]. According to Baumrind, children from authoritative households are more likely to show acceptable social conduct, engage with their peers, and often feel at ease among new people; these children have a greater awareness of how to satisfy others. Higher degrees of academic success have been linked to an authoritative parenting style [10].

The next type of parenting style is authoritarian (high control, low warmth), which is defined by the use of strong parental methods such as physical punishment and yelling by the parent [9]. Authoritarian parents practices lack warmth and communication. These parents lay out concrete expectations [11]. The child obeys the rules that their parents set. Authoritarian parents frequently employ harmful criticism in addition to threatening their children in a harsh and inconsistent manner. These children typically disregard their company and have an innate fear of everything. Additionally, this group of children has poor academic achievement. The children’s conduct in the dental clinic may be connected to all the information presented. It is hypothesized that children who are wary and suspicious are less likely to agree to dental treatments [12, 13].

The third type of parenting style is the permissive pattern (high warmth, low control). Permissive parents don’t forcefully control their children and give them little or no commands or limits to behavior. Studies have indicated that these children are perceived as generally more self-centered, spoiled, and incapable of regulating their impulses; a child with a permissive parenting style may act in a way that makes the dental appointment more difficult, particularly if the child refuses to participate [14].

In 2002, the American Board of Pediatric Dentistry acknowledged that parenting styles had evolved over the years of their practice. Notably, 92% of members believed these changes were negative, and 85% felt they contributed to worse patient behavior [15]. Current research highlights this shift in parenting techniques, particularly the increase in indulgent parenting. This transformation has been linked to diminished children’s behavioral self-regulation, a higher likelihood of dental issues, and reduced parental authority over their children’s behavior [16, 17].

Certain behaviors can extend the period spent on the dental chair or cause a need to apply more advanced behavior management approaches, like general anesthesia and sedation. Parents of today desire to shield their children from suffering or discomfort. Sheller says this is far from the “traditional” parenting methods of applying restrictions and saying “no” [18]. Many parents give less control over their children and are less responsible for the actions they perform [17]. Pediatric dentists should consider the evolving nature of parenting practices to provide each patient with the most effective and efficient treatment.

Although evidence supports a potential correlation between parenting styles and child behavior, few studies discuss this topic in the literature; therefore, this study aimed to investigate the effect of different parenting styles on the child’s behavior on the dental chair [19–21].

The null hypothesis of this study was that there would be no significant effect of different parenting styles on the child’s behavior during the dental visit.

## Methods

### Study design and allocation

The current study was an observational longitudinal study aimed at investigating the correlation between different parenting styles and a child’s behavior during the dental visit. The study was set up and reported in accordance with the STROBE guidelines [22]. Participants were selected from the outpatient clinic, Department of Pediatric Dentistry and Dental Public Health, Faculty of Dentistry, Alexandria University, Egypt.

### Sample size calculation

The minimal sample size was calculated based on a study investigating the effect of parenting style on the choice of proper behavior guidance strategies in Pedodontics. Aminabadi and Farahani (2008) concluded that a child’s reaction to restorative dental procedures is influenced by the nature of the caregiver’s parenting style [23]. Based on their study, a minimum sample of 60 patients is the required sample size for this observational longitudinal study [24, 25] with a power of 80% (accepted β error = 20%) and a 95% level of significance (accepted α error = 5%). Any sample withdrawn from the study will be replaced to maintain the sample size [26]. The sample size was computed according to Charan and Biswas [27]. Online Open Source Epidemiologic Statistics for Public Health were also used to confirm the calculation [28].

### Inclusion criteria

Sixty healthy participants were selected from the Pediatric Dentistry and Dental Public Health Department, Faculty of Dentistry, Alexandria University, Egypt. Only the first child was enrolled in this study to avoid the effect of different birth sequences that might affect the child’s behavior. All participants were aged from 4 to 6 years old (preschoolers) to standardize the child’s cognitive, emotional, and social development and minimize the school peer influence after age six. Participants with no previous dental experience were selected to ensure that the child’s behavior was not affected by any bad previous dental experience. Only class I restorative treatment in one of the primary molars of the participants was treated in the study to standardize the clinical procedure and to minimize the effect of different painful stimuli or different durations of dental procedures. Parents’ consent to participate in the study was provided before the start of treatment.

Parents’ eligibility criteria: only educated parents were selected as a lack of education might affect the manner in which parents deal with their children. Parents’ age ranged from 25 to 40 years old as this age represents the peak age of mental and physical well-being and thus represents the peak capacities of the parents for their child care. Parents enrolled in the study were free of any mental or psychological disturbance that might change their parenting style and disrupt their relationship with their children.

Participants with a history of phobias related to dental settings, any behavioral disorder, or having any caregiver other than their parents were excluded.

### Examiner reliability

The investigator was trained and calibrated by conducting training sessions for the Frankl rating scale of child behavior [29]. The behavior of 10 participants was rated according to the Frankl Rating Scale during clinical examination, and the procedure was repeated after 2 weeks to check intra-examiner reliability. This group of participants was excluded from the study sample. Intra-examiner reliability was evaluated by interclass correlation (ICC) [30].

### Clinical procedures

#### The first dental visit

Throughout the child’s treatment, the parent remained in the operating room passively. The dentist introduced the dental clinic and the examination tools to the child using the tell-show-do technique (TSD) [31]. No treatment was given to build a strong rapport between the child and the dentist [32]. The routine procedures for the first dental visit were carried out, including a comprehensive clinical examination using a mirror and probe, dental prophylaxis “R&D Series Fissured Nova (lmicrly, Turkey)”, and dental radiographs were taken according to the age of the child to ensure a correct diagnosis. Child behavior was assessed using the Frankl scale throughout various dental procedures, and the mean rating scale was subsequently determined at the end of the appointment [29, 33].

#### The second dental visit (after one week)

The second restorative visit was done one week after the examination visit. The routine procedures for class I composite restoration were carried out [34]: the anesthesia administration was explained in simple terminology appropriate to their age. Local anesthesia was applied using Benzocaine Gel 20%. A 27-gauge short needle was used for maxillary infiltration, while a 30-gauge long needle was used for inferior alveolar nerve block anesthesia. Articaine Hydrochloride, 4% with 1:100,000 epinephrine, was used as an anesthetic solution. A rubber dam was then applied, and class I cavity preparation was done using 330 pedo bur with a high-speed turbine and a coolant system; light, intermittent strokes were done while keeping the bur centered within the groove to avoid removing too much tooth structure. The width of the cavity was approximately 1/3 the inter-cuspal distance, and the depth was 0.25 to 0.5 mm in dentin. After complete caries removal, acid etch “Eco-Etch gel was applied for 20 seconds, rinsed and dried, dentin bonding agent was applied and light-cured for 20 seconds, and finally, composite resin was applied, carved, and cured for 40 seconds [35]. The procedure time was assessed in minutes and seconds at the end of the dental procedure.

Participants and parents were told that the first and second visits would be videotaped as a way of motivation to document their good behavior.

#### Child’s behavior assessment

Participants’ behavioral assessment was conducted using the Frankl rating scale (Appendix A) [29] which it is an accurate, simple, non-intrusive method and is considered one of the most widely used systems that are easily integrated into ongoing clinical activities [33].

The child’s behavior was assessed from the recorded videotaped dental visits during different dental procedures: anesthesia administration, rubber dam placement, cavity preparation, and final restoration. The average rating scale was then calculated for each visit: definitely negative, negative, positive, and definitely positive.

All the restorative treatment and the child behavior assessment were performed by the same operator.

### Parental questionnaires: two questionnaires were used in this study

#### Demographic information questionnaire (appendix C) [36]

This questionnaire was used to determine the socio-economic level of the parents, including household income, parental education level, and number of children.

Each item in the questionnaire has different answers with different scores ranging from zero (lowest) to eight (highest). The parents choose different answers with different scores. The total score was calculated by summing the individual scores, and upon this score, the socio-economic level was divided into three categories: high (33.6–48), medium (19.2 to < 33.6), and low (< 19.2).

#### The parenting styles and dimensions questionnaire (PSDQ) (Appendix B) [37]

In this study, the Parenting Styles and Dimensions Questionnaire, designed by Robinson et al., was used to assess the parenting style. It was used because it can be easily understood and answered by the parents. Also, many researchers have proven it to be a reliable and valid tool [37].

The questionnaire was translated into Arabic to be applicable to the Egyptian parents enrolled in the study. This questionnaire was composed of 30 items that were categorized into three groups: authoritative, authoritarian, and permissive parenting styles, which were determined by the way in which parents responded to their children’s behavior [37]. For each issue, a scale ranging from one to six was employed. One represents never; two represents seldom; three represents sometimes; four represents half of the time; five represents frequently; and six represents constantly. The scoring key extends from zero to five.

### Statistical analysis

Data were analyzed using IBM SPSS version 23, Armonk, NY, USA. All qualitative data were presented using frequency and percentage, while the Frankl behavior scale and parenting style were presented mainly using median, minimum, and maximum values. A comparison of the child’s age, operative time, and child’s behavior during 1st and 2nd visits in relation to parenting style was done using the Kruskal Wallis test followed by Dunn’s test with Bonferroni correction. The child’s gender, mother’s education, employment status, SES status, and behavior in relation to parenting style were analyzed using the Chi-Square test. A comparison of Frankl behavior scores at different dental procedures was done using the Friedman test, followed by a post hoc test with Bonferroni correction. A multilevel binomial logistic regression was performed to assess the effect of demographic variables and parenting style on a child’s behavior. All tests were two-tailed, and *p* value was set at 0.05.

## Results

### Demographic distribution

**Fig. 1:**
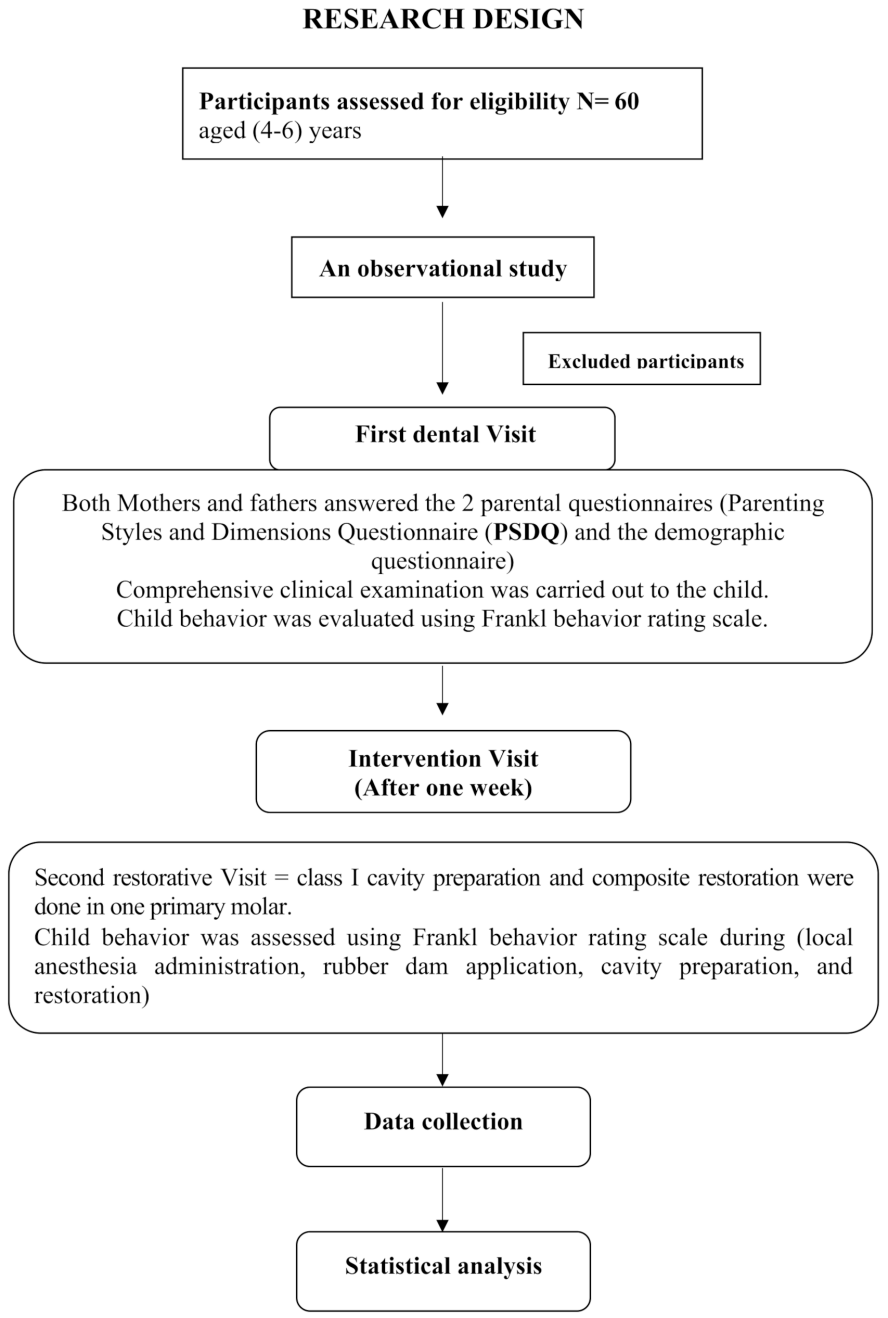
STROBE flow diagram of the study design

**Fig. 2:**
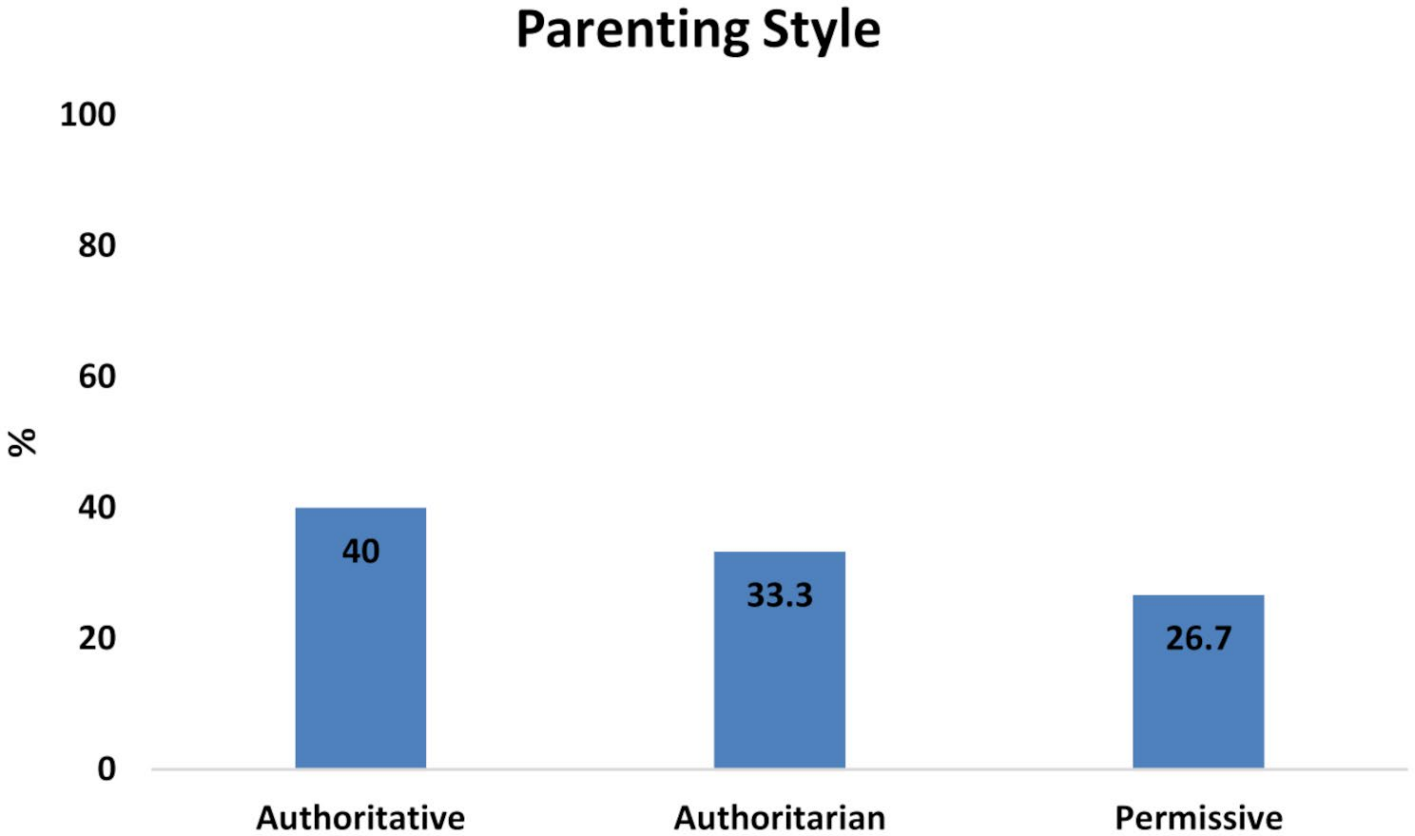
Parenting styles for the study sample

The subject’s recruitment, intervention, and data analysis are illustrated in the STROBE flow diagram (Fig. 1). A total of 60 participants (33 females and 27 males), with a mean age of 5.25 participated in the study (Table 1). The number of authoritative, authoritarian, and permissive parents was 24, 20, and 16, respectively (Fig. 2).

**Table 1:** Demographics of the study sample

Variables		*N* = 60
Age: Mean (SD)		5.25 (0.86)
Gender: *n* (%)	Males	27 (45%)
	Females	33 (55%)
Mother’s education: *n* (%)	Less than secondary school	27 (45%)
	Secondary school	30 (50%)
	University and higher	3 (5%)
Father’s education: *n* (%)	Less than secondary school	22 (36.7%)
	Secondary school	29 (48.3%)
	University and higher	9 (15%)
Mother’s employment: *n* (%)	Yes	31 (51.7%)
	No	29 (48.3%)
Father’s employment: *n* (%)	Yes	59 (98.3%)
	No	1 (1.7%)
Income: *n* (%)	Not enough + loan not repaid	1 (1.7%)
	Not enough + big loan	17 (28.3%)
	Not enough + small loan	27 (45%)
	Enough only	12 (20%)
	Enough and saving	3 (5%)
Computer use: *n* (%)	Never	32 (53.3%)
	Sometimes	13 (21.7%)
	Alot	15 (25%)
Family size: *n* (%)	≥ 7	5 (8.3%)
	6	13 (21.7%)
	5	18 (30%)
	< 5	24 (40%)
Socioeconomic level: *n* (%)	Low	21 (35%)
	Middle	29 (48.3%)
	High	10 (16.7%)

There was no statistically significant difference in gender distribution between the three parenting style types. The number of males in authoritative, authoritarian, and permissive was 11, 9, and 7, respectively, and the number of females was 13, 11, and 9, respectively, with a *p*-value (*P* = 0.992). Regarding the age group, there was a statistically significant difference between authoritarian (5.58) and permissive (4.81) parenting style (*P* = 0.031), but there was no statistically significant difference between authoritative (5.27) and authoritarian (5.58) (*p* = 0.649) nor between authoritative (5.58) and permissive (4.81) (*p* = 0.396). No statistically significant difference was found in mother education, employment status, and socio-economic status between the three parenting style groups (*p* = 0.270, 0.270, 0.061, respectively). No statistically significant difference was found in the father’s education, employment status, and socio-economic status between the three parenting style groups (*P* = 0.362, 0.362, 0.06, respectively).

### Child behavior rating according to different parental styles

The results of the present study showed a statistically significant difference in the Frankl rating scale between the three parenting style groups in both examination and restorative visits:

### Child behavior rating during the examination visit

Children of authoritative parents showed significantly positive behavior (Frankl rating 3) compared to negative behavior (Frankl rating 2) in children of both authoritarian and permissive parents during the following procedures: examination, X-ray, and fluoride application (*P* = 0.007*, *p* = 0.038*, *P* = 0.003* respectively). The overall child behavior during the examination visit showed that children of authoritative parents demonstrated significantly positive behavior compared to negative behavior in children of both authoritarian and permissive parents (*P* = 0.002) (Table 2).

**Table 2:** Child’s behavior according to Frankl scale in relation to parenting style during the first and second visits

		Authoritative	Authoritarian	Permissive	*p* value
		Median (Min–Max)			
1st visit	Examination	3.0 (2.0–4.0)	2.0 (2.0–3.0)	2.0 (2.0–3.0)	0.007*
	X-ray	3.0 (2.0–4.0)	2.0 (2.0–3.0)	2.0 (2.0–3.0)	0.038*
	Fluoride	3.0 (2.0–4.0)	2.0 (2.0–3.0)	2.0 (2.0–3.0)	0.003*
	Overall	3.0 (2.0–4.0)	2.33 (2.0–3.0)	2.33 (2.0–3.0)	0.002*
2nd visit	Anesthesia	3.0 (2.0–4.0)	2.0 (2.0–3.0)	2.0 (2.0–3.0)	0.037*
	Rubber dam	3.0 (2.0–4.0)	2.0 (2.0–3.0)	2.0 (2.0–3.0)	0.006*
	Handpiece	3.0 (2.0–4.0)	2.0 (2.0–3.0)	2.0 (2.0–3.0)	0.007*
	Restoration	3.0 (2.0–4.0)	3.0 (2.0–4.0)	3.0 (2.0–4.0)	0.001*
	Overall	3.0 (2.0–4.0)	2.25 (2.0–3.25)	2.25 (2.0–3.25)	0.004*

*Statistically significant difference at *p* value ≤ 0.05

### Child behavior during the restorative visit

Children of authoritative parents showed significantly positive behavior (Frankl rating 3) compared to negative behavior (Frankl rating 2) in children of both authoritarian and permissive parents during the following procedures: anesthesia administration, rubber dam application, cavity preparation, and restoration (*P* = 0.037*, *p* = 0.006*, *P* = 0.007*, *P* = 0.001*, respectively). The overall child behavior on the dental chair showed that children of authoritative parents showed significantly positive behavior compared to negative behavior in children of both authoritarian and permissive parents (*P* = 0.004) (Table 2).

### The effect of parental style on the time of the dental procedure

The authoritative parenting style showed significantly less operative time (13.83 min) compared to both the authoritarian (26.61 min) and permissive groups (26.25 min) (*P* = 0.001, 0.008, respectively). There was no statistically significant difference in the operative time between authoritarian and permissive groups with (*P* = 1.00) (Table 3).

**Table 3:** Operative time according to parenting styles for the study sample

	Authoritative (*n* = 24)	Authoritarian (*n* = 20)	Permissive (*n* = 16)
Mean (SD)	16.20 (9.18)	25.46 (11.06)	26.52 (10.82)
Median (Min-Max)	11.45 (8.02–34.45)	28.01 (9.03–40.41)	30.01 (8.47–38.44)
*P* value	0.007*		
Pairwise comparison	Authoritative vs. Authoritarian = 0.018* Authoritative vs. Permissive = 0.030* Authoritarian vs. Permissive = 1.00		

*Statistically significant difference at *p* value ≤ 0.05

According to the results of the present study, the null hypothesis was rejected as the findings showed that the parenting style had a great influence on the child’s behavior on the dental chair.

## Discussion

Different factors affect the behavior of children on the dental chair; one of the most important factors is the parenting style, as it has a significant impact on the child’s social, emotional, and cognitive development and thus has a direct impact on the child’s behavior on the dental chair [3, 38]. Recently, parenting styles have gained great interest from pediatric dentists due to their significant influence on the child’s personality development [39]. However, up till now, few researches have been found in the literature addressing this topic [23, 40]. Therefore, this study was done to investigate the correlation between parenting styles and a child’s behavior on the dental chair.

After observing the behavior of the children in both the examination visit and the restorative visit, the findings of this study suggested that children with authoritative parents showed significantly positive behavior (Frankl rating 3) compared to those of authoritarian and permissive parents who showed negative behavior on the dental chair (Frankl rating 2). This result was consistent with the results of most investigations, including those by Aminabadi et al. [23, 41] and Howenstein et al. [17]. The possible explanation is that children who grow up in authoritative homes are happier, have better social skills, and have better emotional regulation and coping mechanisms [42]. On the other hand, the negative behavior of the children of authoritarian and permissive parenting styles presented in this study was either due to their lack of confidence in people, fearfulness, and inability to interact socially or due to the overindulgence resulting in spoiled behavior and refusal to treatment [43, 44], The result of the present study was inconsistent with Krikken et al. [45, 46] who concluded that there were no differences in children’s behavior between various parenting styles. Our justification for the different findings might be attributed to the variations in the chosen age groups and prior dental exposure. In the present study, we selected a small age range group from 4 to 6 years (preschoolers) to standardize the child’s development as much as possible regarding cognitive, emotional, and social characteristics. Also, we selected children with no previous dental experience to investigate the real child behavior, either positive or negative, while, in the Krikken et al. study, a wide age group was enrolled from 4 to 12 years, resulting in different stages of child development [39, 40]. In the school years starting from 6, peers’ and teachers’ attitudes might have a great influence on the child’s behavior, obscuring the parental style’s influence on the child [47]. Moreover, children with bad dental experience were included in the sample of the Krikken et al. study; therefore, the learned fear they experienced from the bad dental experience might obscure the influence of parenting practices on the real child’s behavior [48].

The study findings also demonstrated that children with the authoritative parenting style recorded significantly shorter dental procedure times than those with authoritarian and permissive parenting styles. Our justification for these findings is that the children of the authoritative parents showed better cooperation and acceptance of the different dental procedures and dentist instructions; thus, basic management techniques such as tell, show, do, and distraction by video cartoons were used. In contrast, the children of authoritarian and permissive parenting groups exhibited delaying tactics, crying, refusal to open their mouth, and sometimes even temper tantrums were encountered, so additional behavior management techniques were used as modeling, parental presence/absence, and voice control, thus lengthening the dental procedure.

## Conclusion

The parental style had a significant effect on the child’s behavior on the dental chair. The children of authoritative parents showed positive behavior (Frankl rating 3), while the children of both authoritarian and permissive parents showed negative behavior (Frankl rating 2).

Operative time spent on the dental chair was significantly influenced by the parenting style. Children of authoritative parents needed significantly less operative time than the authoritarian and permissive groups, which is a highly significant and beneficial factor for both the dentist and the child to provide comfortable, pleasant, and effective dental treatment.

### Recommendation

1. More studies are still needed to study the influence of parenting styles on children’s behavior in different age groups.
2. Parent awareness through health education and encouraging an authoritative attitude would make a great difference not only in the child’s behavior on the dental chair but also in his interaction with different aspects and challenges of life.

## Electronic supplementary material

Below is the link to the electronic supplementary material.

Appendix A: Frankl Scale

Appendix B: The Parenting Styles and Dimensions Questionnaire (PSDQ)

Appendix C: Demographic questionnaire

## Data Availability

The datasets developed and analyzed for the present study were found in the patient’s records at the Pediatric Dentistry Department, Faculty of Dentistry, Alexandria University, Egypt. These datasets were utilized under a license for publication purposes. There are limitations on the public’s ability to access these data. However, upon request and with permission from the Pediatric Dentistry Department, Faculty of Dentistry, Alexandria University, data from the corresponding author are available.

## Abbreviations

ICC: Interclass correlation
PSDQ: The Parenting Styles and Dimensions Questionnaire
SES: Socio-economic status
TSD: Tell-show, and do
