# Supplementary material for: The effect of different parenting styles on the child behavior during the dental visit: observational longitudinal study
Source: BMC Oral Health. 2025 Mar 5;25:342. doi: 10.1186/s12903-025-05659-2 (PMC11883977; doi:10.1186/s12903-025-05659-2)
Supplement: Supplementary file 1 — Appendix A: Frankl Scale [file 12903_2025_5659_MOESM1_ESM.docx]

**Appendix A: Frankl Scale**

| 1) -/- Definitely Negative: | Refusal of treatment, forceful crying, fearfulness, or any other evidence of extreme negatism. |
| --- | --- |
| 2) – Negative: | Reluctance to accept treatment, uncooperative, some evidence of negative attitude but not pronounced (sullen, withdrawn). |
| 3) + Positive: | Acceptance of treatment; cautious behavior at times; willingness to comply with the dentist, at times with reservation, but patient follows the dentist’s directions cooperatively |
| 4) +/+ Definitely positive: | Good rapport with the dentist, interest in the dental procedures, laughter, and enjoyment. |
