# Supplementary material for: The effect of different parenting styles on the child behavior during the dental visit: observational longitudinal study
Source: BMC Oral Health. 2025 Mar 5;25:342. doi: 10.1186/s12903-025-05659-2 (PMC11883977; doi:10.1186/s12903-025-05659-2)
Supplement: Supplementary file 2 — Appendix B: The Parenting Styles and Dimensions Questionnaire (PSDQ) [file 12903_2025_5659_MOESM2_ESM.docx]

**Appendix B: The Parenting Styles and Dimensions Questionnaire (PSDQ)**

1. I am responsible for my child’s feelings and needs

Never 1 2 3 4 5 6 Always

2. I take my child’s wishes into consideration before I ask him/her to do something

Never 1 2 3 4 5 6 Always

3. I explain to my child how I feel about his/her good/bad behavior

Never 1 2 3 4 5 6 Always

4. I encourage my child to talk about his/her feelings and problems

Never 1 2 3 4 5 6 Always

5. I encourage my child to freely “speak his/her mind”, even if he/she disagrees with me

Never 1 2 3 4 5 6 Always

6. I explain the reasons behind my expectations

Never 1 2 3 4 5 6 Always

7. I provide comfort and understanding when my child is upset

Never 1 2 3 4 5 6 Always

8. I compliment my child

Never 1 2 3 4 5 6 Always

9. I consider my child’s preferences when I make plans for the family (e.g., weekends away and holidays)

Never 1 2 3 4 5 6 Always

10. I respect my child’s opinion and encourage him/her to express them

Never 1 2 3 4 5 6 Always

11. I treat my child as an equal member of the family

Never 1 2 3 4 5 6 Always

12. I provide my child, the reasons for the expectations I have for him/her

Never 1 2 3 4 5 6 Always

13. I have warm and intimate times together with my child

Never 1 2 3 4 5 6 Always

1. When my child asks me why he/she has to do something I tell him/her it is because I said so, I am your parent, or because that is what I want

Never 1 2 3 4 5 6 Always

2. I punish my child by taking privileges away from him/her (e.g., TV, games, visiting friends)

Never 1 2 3 4 5 6 Always

3. I yell when I disapprove of my child’s behavior

Never 1 2 3 4 5 6 Always

4. I explode in anger towards my child

Never 1 2 3 4 5 6 Always

5. I spank my child when I don’t like what he/she does or says

Never 1 2 3 4 5 6 Always

6. I use criticism to make my child improve his/her behavior

Never 1 2 3 4 5 6 Always

7. I use threats as a form of punishment with little or no justification

Never 1 2 3 4 5 6 Always

8. I punish my child by withholding emotional expressions (e.g., kisses and cuddles)

Never 1 2 3 4 5 6 Always

9. I openly criticize my child when his/her behavior does not meet my expectations

Never 1 2 3 4 5 6 Always

10. I find myself struggling to try to change how my child thinks or feels about things

Never 1 2 3 4 5 6 Always

11. I feel the need to point out my child’s past behavioral problems to make sure he/she will not do them again

Never 1 2 3 4 5 6 Always

12. I remind my child that I am his/her parent

Never 1 2 3 4 5 6 Always

13. I remind my child of all the things I am doing and I have done for him/her

Never 1 2 3 4 5 6 Always

1. I find it difficult to discipline my child

Never 1 2 3 4 5 6 Always

2. I give into my child when he/she causes a commotion about something

Never 1 2 3 4 5 6 Always

3. I pamper my child

Never 1 2 3 4 5 6 Always

4. I ignore my child’s bad behavior

Never 1 2 3 4 5 6 Always
