# Supplementary material for: The effect of different parenting styles on the child behavior during the dental visit: observational longitudinal study
Source: BMC Oral Health. 2025 Mar 5;25:342. doi: 10.1186/s12903-025-05659-2 (PMC11883977; doi:10.1186/s12903-025-05659-2)

**Appendix C: Demographic questionnaire**

1. Translation to Arabic.
2. Back translation to English.
3. Comparison of the new back-translated English version with the original English version.


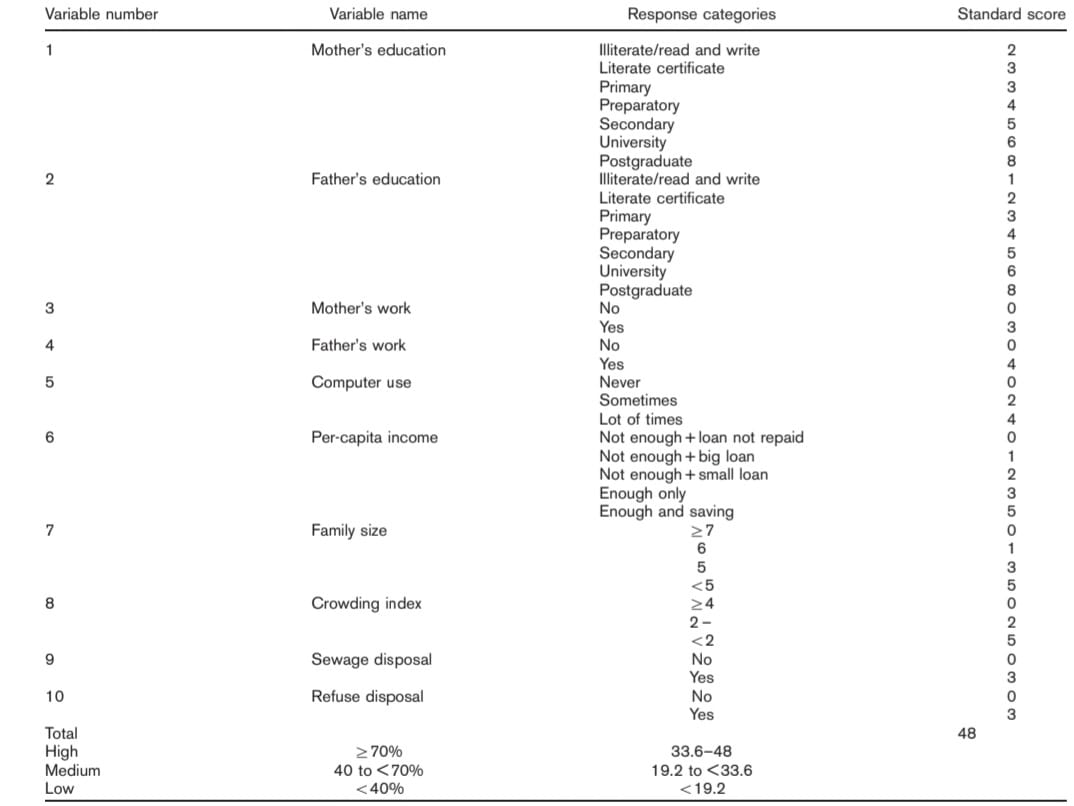

Supplement: Supplementary file 3 — Appendix C: Demographic questionnaire [file 12903_2025_5659_MOESM3_ESM.docx]
